# A Bounded Integer Model for Rating and Composite Scale Data

**DOI:** 10.1208/s12248-019-0343-9

**Published:** 2019-06-06

**Authors:** Gustaf J. Wellhagen, Maria C. Kjellsson, Mats O. Karlsson

**Affiliations:** Pharmacometrics Research Group, Department of Pharmaceutical Biosciences, Uppsala University, Box 591, 751 24 Uppsala, Sweden

**Keywords:** Bounded integer model, Categorical data, Composite scale, Nonlinear mixed-effects modelling, Probit regression, Rating scale

## Abstract

Rating and composite scales are commonly used to assess treatment efficacy. The two main strategies for modelling such endpoints are to treat them as a continuous or an ordered categorical variable (CV or OC). Both strategies have disadvantages, including making assumptions that violate the integer nature of the data (CV) and requiring many parameters for scales with many response categories (OC). We present a method, called the bounded integer (BI) model, which utilises the probit function with fixed cut-offs to estimate the probability of a certain score through a latent variable. This method was successfully implemented to describe six data sets from four different therapeutic areas: Parkinson’s disease, Alzheimer’s disease, schizophrenia, and neuropathic pain. Five scales were investigated, ranging from 11 to 181 categories. The fit (likelihood) was better for the BI model than for corresponding OC or CV models (ΔAIC range 11–1555) in all cases but one (∆AIC − 63), while the number of parameters was the same or lower. Markovian elements were successfully implemented within the method. The performance in external validation, assessed through cross-validation, was also in favour of the new model (ΔOFV range 22–1694) except in one case (∆OFV − 70). A residual for diagnostic purposes is discussed. This study shows that the BI model respects the integer nature of data and is parsimonious in terms of number of estimated parameters.

**Electronic supplementary material:**

The online version of this article (10.1208/s12248-019-0343-9) contains supplementary material, which is available to authorized users.

## INTRODUCTION

Many clinical trial endpoints are measured with rating scales or composite scales. Rating scales, such as the Likert scale, are typically based on a single assessment or question (e.g. “How much pain do you feel?”) while composite scales consist of several assessments or questions that generate a total score. The nature of such scale-based data is complex, and there is no fully satisfying modelling approach when the number of possible categories is many.

The most common strategy is to treat the outcome as a continuous variable (CV), while knowing that the underlying data is of a categorical or integer nature. This poses a problem especially at the scale boundaries, where the residual error can give predictions outside the expected range. Logistic transformation or beta regression can constrain the variable, but then a model can only predict the extreme values asymptotically (1). Also, the continuous variable needs to be rounded and/or truncated to simulate real-life-like examples (2).

Another approach is to treat the outcome as an ordered categorical variable (OC), which instead requires as many parameters, save one, as the number of categories only to capture the baseline characteristics. More observations are also required to estimate these parameters as the number of categories increases. Another drawback of OC models is that they cannot simulate outside the range of observations, whether it be interpolation or extrapolation.

Latent variable models for categorical data have been used for a long time (3). Previous work has mostly focused on scales with only a few categories (< 10) (e.g. no-mild-moderate-severe) but not on rating scales or composite scales with a larger number of possible categories (4–7). New methods have also been suggested to deal with ordinal data within nonlinear mixed-effects modelling in a parsimonious way (8). Probit regression for bounded outcome scores (BOS) for composite scale data is a promising concept which has been described for one data set previously (9).

Here, we present the bounded integer (BI) model for modelling rating and composite scale data aiming for parsimony, while respecting the integer nature of the data. Using previously published data, we compare the bounded integer model with OC and CV models for situations where the number of categories is high (11 for rating scale and > 70 for composite scales). We also show how Markovian elements can be implemented in these models.

## METHODS

### The Bounded Integer Model

For a scale with *n* categories, the area under a standard normal distribution with a mean of 0 and variance of 1 (*N*(0,1)) is divided into *n* equal-sized areas through *n* − 1 cut-off values via the probit (quantile function of the standard normal distribution): *Z*_1/*n*_ to *Z*_(1 − *n*)/*n*_.

A function of fixed effects (*θ*) and random effects for an individual *i* (*η*_*i*_), time and covariates (*X*_*i*_), *f*(*θ*,*η*_*i*,*f*_*,t*,*X*_*i*,*f*_) with variance function *g*(*σ*,*η*_*i*,*g*_,*t*,*X*_*i*,*g*_) is used together with the *Z*-values to estimate the probability of each category. The two functions define a normal distribution: *N*(*f*(*θ*,*η*_*i*,*f*_,*t*,*X*_*i*,*f*_), *g*(*σ*,*η*_*i*,*g*_,*t*,*X*_*i*,*g*_)). Formally, the probability for the *k*th category (*P*_*i*,*j*_(*k*)) is defined in Eq. 1:

1 $$ {P}_{i,j}(k)=\phi \left(\frac{Z_{\frac{k}{n}}-f\left(\uptheta, {\upeta}_{i,f},t,{X}_{i,f}\right)\ }{g\left(\upsigma, {\upeta}_{i,g},t,{X}_{i,g}\right)\ }\right)-\phi \left(\frac{Z_{\frac{k-1}{n}}-f\left(\uptheta, {\upeta}_{i,f},t,{X}_{i,f}\right)\ }{g\left(\upsigma, {\upeta}_{i,g},t,{X}_{i,g}\right)\ }\right) $$

where *φ* is the cumulative distribution function of the normal distribution; in other words, the probability of each score is defined as the area under the latent variable defined function within the interval given by the cut-offs. For the first category (*k = 1*), Eq. 1 collapses into Eq. 2:

2 $$ {P}_{i,j}(1)=\phi \left(\frac{Z_{\frac{1}{n}}-f\left(\uptheta, {\upeta}_{i,f},t,{X}_{i,f}\right)\ }{g\left(\upsigma, {\upeta}_{i,g},t,{X}_{i,g}\right)\ }\right) $$

since this is the cumulative distribution in the interval [− ∞,*Z*_1/*n*_), and for the last category (*k = n*) into Eq. 3:

3 $$ {P}_{i,j}(n)=1-\phi \left(\frac{Z_{\frac{n-1}{n}}-f\left(\uptheta, {\upeta}_{i,f},t,{X}_{i,f}\right)\ }{g\left(\upsigma, {\upeta}_{i,g},t,{X}_{i,g}\right)\ }\right) $$

since this is the cumulative distribution in the interval [*Z*_(*n*-1)/*n*_,∞]. A formal definition of the likelihood under this model is provided in the supplemental equations.

### Data Sets

**Fig. 1:**
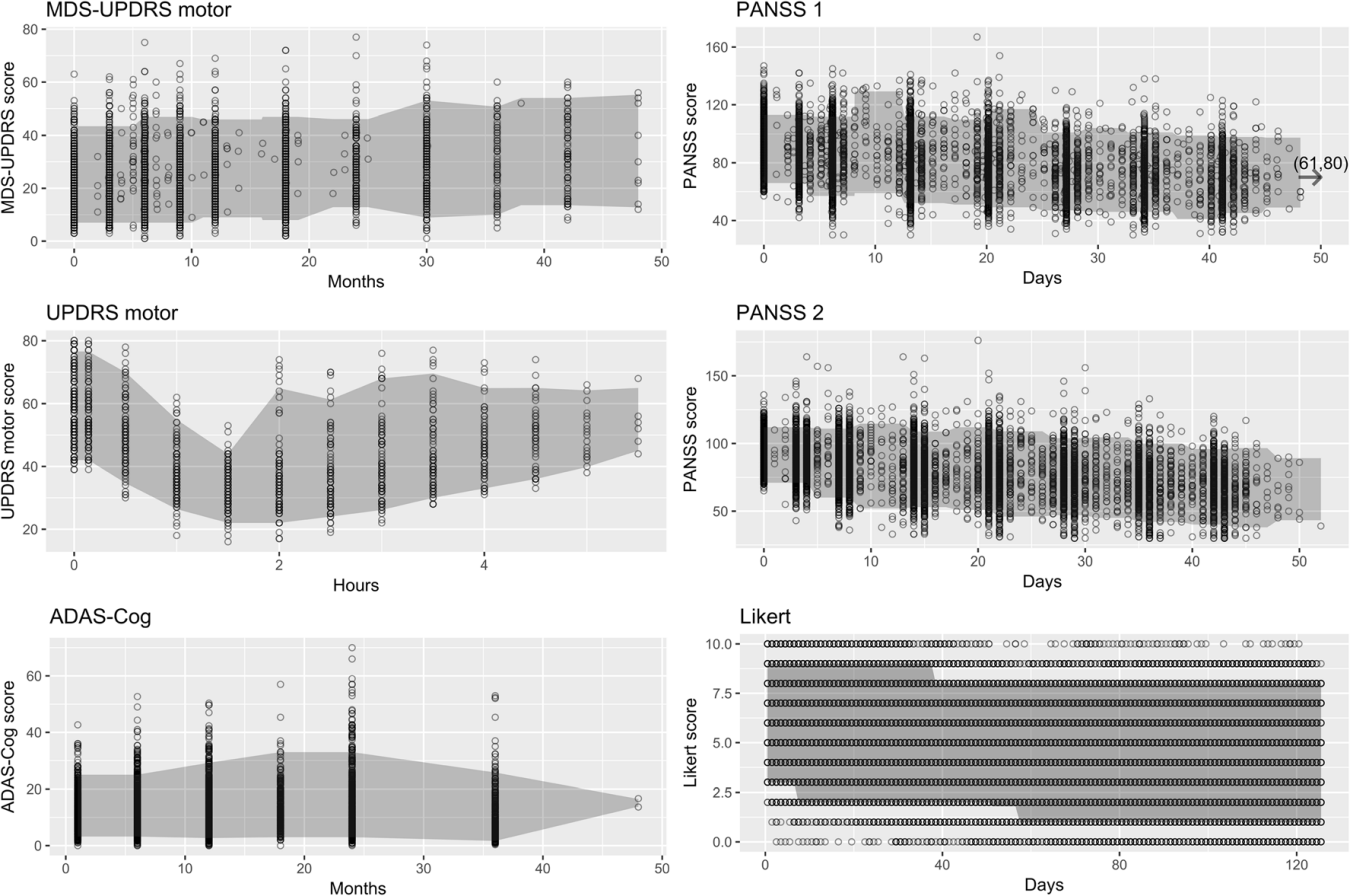
Score time course and 90% prediction intervals for the investigated data sets. MDS-UPDRS, Movement Disorder Society-Unified Parkinson’s Disease Rating Scale; UPDRS, Unified Parkinson’s Disease Rating Scale; ADAS-Cog, Alzheimer’s Disease Assessment Scale-Cognitive; PANSS, Positive and Negative Syndrome Scale

Several data sets were used in the investigation, representing both rating scale data (Likert, where patients were asked to rate their pain with an integer between 0 and 10) and composite scale data (all others). A visual representation of the data sets is shown in Fig. 1. The data sets varied in disease area, number of categories, number of observed categories and number of observations as shown in Table I.

**Table I:** A summary of data set characteristics and references

Disease	Scale	Categories	Observed range (theoretical)	No. of patients	No. of Obs	Reference data	Reference models
Parkinson’s disease	MDS-UPDRS motor (10)	133	1–77 (0–132)	428	2720	(11)	(12)
Parkinson’s disease	UPDRS motor (13)	109	16–80 (0–108)	19	946	(12)	(12)
Alzheimer’s disease	ADAS-Cog (14)	71	0–70 (0–70)	817	3594	(15)	(15)
Schizophrenia	PANSS (16)	181	30–176 (30–210)	1323	7728	(17)	(17)
Schizophrenia	PANSS (16)	181	30–167 (30–210)	1292	8520	(18)	(18)
Neuropathic pain	Likert^a^	11	0–10 (0–10)	231	22,492	(19)	(19,20)

*MDS-UPDRS, Movement Disorder Society-Unified Parkinson’s Disease Rating Scale; UPDRS, Unified Parkinson’s Disease Rating Scale; ADAS-Cog, Alzheimer’s Disease Assessment Scale-Cognitive; PANSS, Positive and Negative Syndrome Scale*

^a^ *Rating scale*

### Implementation of the Bounded Integer Model

The rating scale data from the 11-category Likert neuropathic pain scale had previously been modelled with both an OC (20) and a CV (19) approach. Both these models had elements for serial correlation (Markov or autoregressive). The BI model was thus implemented with a Markov element. To achieve this, an additional Markov model component was implemented as described in Eq. 4:

4 $$ {P}_{i,j}\left(k|{Y}_{i,j-1}=k\right)=\frac{P_{k,i,j}+ PM}{1+ PM} $$

where *Y*_*i*,*j-*1_ is the observation and *P*_*k*,*i*,*j*_ is the probability of a score *k* for individual *i* at time *j*. If *Y*_*i,j*_ and *Y*_*i,j-1*_ are different, the expression is instead as in Eq. 5:

5 $$ {P}_{i,j}\left(k|{Y}_{i,j-1}\ne k\right)=\frac{P_{k,i,j}}{1+\mathrm{PM}} $$

The parameter PM(*θ*,*η*_*i*,*PM*_,*t*,*X*_*i*,*PM*_), constrained to be non-negative, provide when positive a higher probability that an observation has the same value as the previous observation in time, compared with that of the predictions by *f*() and *g*() alone. An exponentially distributed random effect was used to implement interindividual variability in PM.

For the cases where a CV model was available, it was used as a reference model. Full details on these models and the data collection process can be found in the respective publication. A BI model was then implemented with the same structural components and covariates as the reference model. The parameterization could not be identical, as the BI and CV models have a different basic structure, but the number of estimated parameters was made to be the same and the implemented relations qualitatively similar.

In one case (MDS-UPDRS), no CV model was available for the data in questions, and both BI and CV models were constructed with baseline, linear disease progression and a symptomatic drug effect, as indicated from the item response model in the original analysis (10). Interindividual parameter variability was introduced in all three structural model parameters. The NONMEM model file for the final BI model is provided in supplemental code 2.

### Goodness-of-Fit Metrics

The Akaike information criterion (AIC) was used to compare goodness of fit between models. For a model with *m* parameters to estimate, AIC is computed via the objective function value (OFV) as:

6 $$ \mathrm{AIC}=\mathrm{OFV}+2m $$

Thus, for models with the same number of parameters, the difference in AIC or OFV is the same. This was the case for all comparisons, save the comparison between OC and BI models for the Likert data.

### Pearson Residual: a Probability Weighted Residual

For categorical data, residuals do not represent a direct link between the model and the data, as it does for continuous variable. Also, the choice of residual to use for facilitating identification of outliers and model misspecification is not as straightforward. For the BI model, we use the Pearson residual for categorical data (PWRES) (3) for model diagnostic purposes:

7 $$ {\mathrm{PPRED}}_{i,j}={P}_{i,j}(1)\times 1+{P}_{i,j}(2)\times 2+\dots ={\sum}_{k=1}^n{P}_{i,j}(k)\times k $$

8 $$ {\mathrm{SDPRED}}_{i,j}=\sqrt{\sum_{k=1}^n{P}_{i,j}(k)\times {\left(k-{\mathrm{PPRED}}_{i,j}\right)}^2} $$

9 $$ {\mathrm{PWRES}}_{i,j}=\frac{{\mathrm{DV}}_{i,j}-{\mathrm{PPRED}}_{i,j}}{{\mathrm{SDPRED}}_{i,j}} $$

where the *i*th individual’s *j*th observation has response DV_*i*,*j*_, weighted prediction PPRED_*i*,*j*_, standard deviation SDPRED_*i*,*j*_ and weighted residual PWRES_*i*,*j*_. The expected mean and variance of PWRES are approximately 0 and 1.

### Cross-validation

The performance in external validation of the models was investigated through cross-validation (21–25), where the data was split into five equal-size sets. Model parameters were estimated on four-fifth (80%) of the sets, and the resulting parameters, without re-estimation, were used in evaluating the goodness of fit, using OFV as metric, to the fifth (20%), test data, set. This process was repeated five times, one for each set left out. The OFVs for these five sets were then added and used as a measure of performance to data which was not used in the parameter estimation—the lower the cross-validated OFV, the better the performance. Such a metric is a global one and captures the likelihood with which a model can predict data which was not used for the parameter estimation. As no parameters are estimated based on the new data, there is no need to take into account the size of the model when comparing such out-of-sample OFVs.

### Software

Nonlinear mixed-effects modelling was performed with NONMEM version 7.3 (26), executed through PsN version 7.4 (27). Graphics were made with R (28). The Laplace estimation method, with interaction for the CV models, was used for all model evaluations.

## RESULTS

The BI model had fewer parameters compared with the published implementation of an ordered categorical model for the Likert data set (13 and 18, respectively). The ∆AIC was 1555 in favour of the BI model. When ∆OFV was calculated from cross-validated analyses of the two models, the difference was 1694 in favour of the BI model. When the analysis was performed without Markov elements in either the BI or CV model, the ∆AIC was 810 in favour of the BI model.

**Fig. 2:**
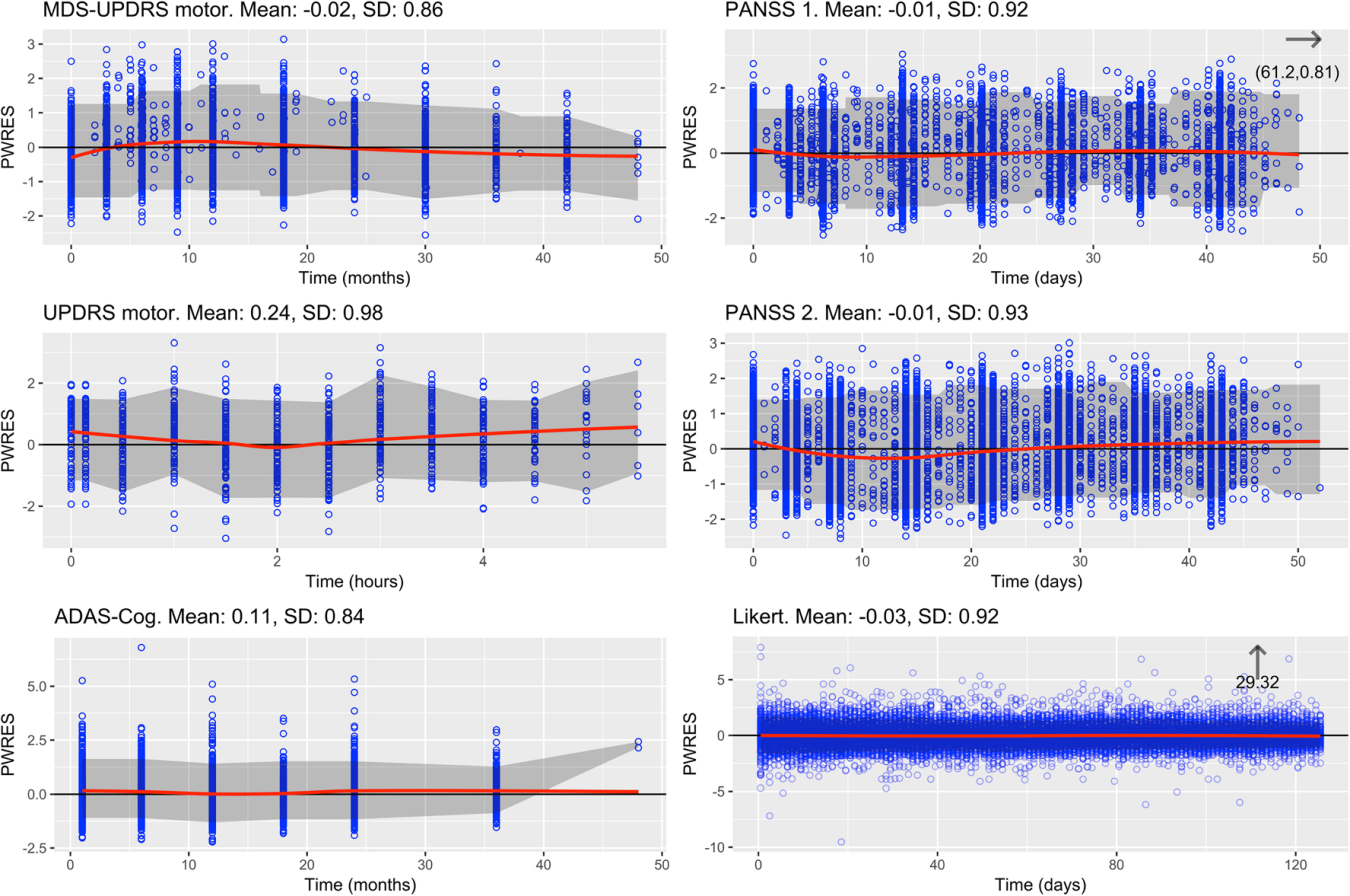
PWRES *vs.* time. MDS-UPDRS, Movement Disorder Society-Unified Parkinson’s Disease Rating Scale; UPDRS, Unified Parkinson’s Disease Rating Scale; ADAS-Cog, Alzheimer’s Disease Assessment Scale-Cognitive; PANSS, Positive and Negative Syndrome Scale

The fit to the Likert data for the previously published CV model displayed an AIC value which was 1945 higher than the corresponding BI model with the same number of parameters. Pearson residuals for the BI model are illustrated in Fig. 2. NONMEM control stream for the BI model is found in supplemental code 2.

The goodness of fit of the BI and corresponding CV model to different data sets with composite scale data is shown in Table II. The BI and CV models in these examples had the same number of estimated parameters. The parameter estimates of the BI models are shown in supplemental table 1. For all the seven BI models, the incorporation of interindividual variability in the *g*() function improved the goodness of fit, with decreases in the OFV ranging from 57 to 4446 (not shown).

**Table II:** Fit to different composite scale data sets for bounded integer and continuous variable models

Disease	Scale	No. of parameters CV = BI	CV AIC	∆AIC CV-BI	∆Cross-validated OFV CV-BI
Parkinson’s disease	MDS-UPDRS motor	14	18,539	74	61
Parkinson’s disease	UPDRS motor	16	5631	62	84
Alzheimer’s disease	ADAS-Cog	11	20,358	729	729
Schizophrenia	PANSS^a^	17	56,178	− 63	− 70
Schizophrenia	PANSS^b^	15	61,575	11	22
Neuropathic pain	Likert^c^	18^d^ *vs.* 13^e^	48,938	1555	1694

*∆AIC, difference in Akaike information criterion; CV, continuous variable; BI, bounded integer; ∆cross-validated OFV, difference in cross-validated objective function value; MDS-UPDRS, Movement Disorder Society-Unified Parkinson’s Disease Rating Scale; UPDRS, Unified Parkinson’s Disease Rating Scale; ADAS-Cog, Alzheimer’s Disease Assessment Scale-Cognitive; PANSS, Positive and Negative Syndrome Scale*

^a^ *Reference 17*

^b^ *Reference 18*

^c^ *Rating scale*

^d^ *Ordered categorical model*

^e^ *Bounded integer model*

Figure 2 displays residual analyses for composite score data. For all the data sets, it has a mean value close to 0 and a variance close to 1. There is a slight trend in some residuals, such as for the UPDRS BI model, indicating that the model structure could be improved.

## DISCUSSION

### Bounded Integer *Versus* Ordered Categorical Model

The BI model described the Likert pain scale data better than the model treating the data as OC, both in fit (∆AIC = 1555) and external validation (cross-validated ∆OFV = 1694). This is the only comparison where there is a difference in the number of parameters between the BI model and the model used for comparison. In this case, there were 5 fewer parameters in the BI model. Thus, a disadvantage of the OC model is that it requires many parameters which make it less suitable for scales with many categories. As the number of categories increases, the OC model will also require more observations to support these parameters. A related problem is that with the OC model, the probability of categories that are not present in the data cannot be estimated. Either the probability of such scores needs to be fixed to zero or individual categories need to be merged into groups. The BI approach handles these probabilities implicitly, making it possible to predict and simulate scores not present in a given data set. The BI model assumes that all scores that make up a scale are possible and also that the probability of a non-extreme score is larger than at least one of the nearest adjacent scores, so that *P*_*i*, *j*_(*k*) > min(*P*_*i*, *j*_(*k* − 1), *P*_*i*, *j*_(*k* + 1)). This inherent assumption could pose a problem if the distribution of scores is for some reason not unimodal, e.g. if 5 and 7 are much more common than 4, 6 and 8. Such data have been observed when pain intensity has been captured using pictures with different face expressions (29). While similar problems may occur when rating scales use verbal expressions to identify categories, one can assume that there is less risk when, as in the present case, pain intensity response is solicited directly using a numerical scale, where patients identify the numerical category they associate with their present pain intensity.

At the boundaries of the scale, responses may be different, containing different types of information. For the Likert pain rating scale, there are numbers from 0 to 10, where 0 represents “no pain” and 10 “the worst pain imaginable”. It is reasonable that patients rate the step size between 1 and 2 as equal to that between 6 and 7. In this example, there could be an aversion to responding 10, and in many other studies, the extreme values have deviant probabilities. For OC models, this is not a problem, as each category is modelled independently of the others. However, as discussed previously, the latent variable distribution assumes a continuous underlying function to estimate the probabilities. For the same reason as above, this type of data may need further parameters or a different latent variable distribution to fit well with the BI model.

The BI model is similar to the BOS model described by Hu *et al.* (9) in the parsimonious approach and the use of probit regression. In their work, they additionally investigated different link functions, or transformations of the outcome, although only for one scale and with one example. Their suggestion is to transform one or either side of the distribution to achieve a normal-appearing distribution. Flexible transformations such as the discretized beta distribution suggested by Ursino and Gasparini (8) are also possible. However, this was outside the scope of this work.

The reason that the BI model described the Likert pain data better than the OC model appeared to be related to the presence of a random effect in *g*(), the BI model variability function. The effect was generally well described (30) (relative standard error < 30%, see supplemental table 1). Without such a random effect, the fit for the BI model was no longer superior to that of the OC model. This interindividual variability in *g*() was formulated as an exponential distribution. It predicts that individuals differ in the consistency with which they report the daily pain scores. A similar type of variability cannot be introduced into the OC model with a single random effect. Rather, it would require a random effect per category, hence increasing the model size considerably.

### Markov Modelling

The implementation of first-order Markovian elements used in the OC model here assumes a higher probability of the same score as the one previously observed. Indeed, it predicts that, if two observations were made in very close proximity in time, the second would have the same score as the first. For the BI model, there are two components to the probability, one given by *f*() and *g*() and the second by the score of the previous observation. The parameter PM estimates the balance between these two. Hence, this BI model implementation can elevate the probability of subsequent same-score observations without making very different scores of two adjacent observations having very low probability. Data with strong Markovian properties often display a small portion of data that makes large jumps between scores of adjacent observations. This feature in the BI model appears to better handle such observations, and the improvement in fit was larger for the BI model than the OC model when Markov elements were included. In both models, the Markovian feature attenuates with time; that is adjacent same-score observations become more probable as the time from study start increases.

### Bounded Integer *Versus* Continuous Variable Models

The BI model described the Likert pain scale data better than the corresponding CV model with the same number of estimated parameters. All scores from 0 to 10 were present in the data, and as described previously, the error in the CV model is not optimal towards the extreme scores of the scale. A CV model might predict values outside the scale boundaries or, if, e.g. logistic transformation or beta regression is used, will only predict the boundaries asymptotically. This model misspecification and the fact that CV models are not treating the data as integers are potential explanations to why the BI model was superior. On the other hand, the CV models can be estimated using the first-order conditional estimation methods which are often both faster and more robust than the Laplacian method.

The models were optimized for CV analysis. Improving the *g*() function, corresponding to the residual model structure in a CV model, could benefit the BI approach even further, which was tested for all models. In all cases, there was a significant drop in AIC when adding a more complex residual structure, for example different variability magnitude at different time points (results not shown).

When investigating the parameter estimates and their uncertainty of the BI models (see supplemental table 1), some parameters with high uncertainty seemed superfluous, e.g. hospitalization for the PANSS 1 data. Upon removal of this parameter, the fit was not significantly worse (results not shown). For all BI models except the Likert and UPDRS, the model could be simplified by removing some parameter or correlation without significant penalty to the fit. This further supports the idea that the CV model structures are not optimized for BI analysis. Further development with a significantly better fit with additional components or model reduction with a comparable fit can be achieved.

### Scale Range and Variance

For both Parkinson’s disease data sets (UPDRS and MDS-UPDRS), the motor scores were well below the maximum value, and the minimum value observed was not 0. For the two PANSS data sets, the maximal observed scores were well below the maximum scale value. The BI model was implemented with as many categories as possible scores, as this would theoretically allow extrapolation beyond the observed score range, while still restrict values to those possible. While this is an attractive feature, it could be hypothesised that a scale restricted to the observed range may provide a better fit to the data. However, when maximally restricted BI models were used to analyse the data, the quality of the fit was similar to using the full range. As we see no benefit of restricting possible scores to the observed range, we recommended to implement the BI model with the full range of the scale in question.

The variance function, *g*(), is of high interest and its interpretation is not straightforward. For a given number of categories, a smaller value would indicate a more predictive model. We would typically expect values of *g*() that are considerably below one; as with a *g*() equal to one, all scores are equally likely, given a *f*() of zero. However, for scales where most observations are at extremes, values higher than one may be anticipated. We have not encountered such scales, and such a scale feature is likely to be avoided in the design of a rating or composite scale. A factor that will play a role in the value of *g*() is the scale range. If the observed range of scores only occupy a fraction of the theoretical scale range used, the *g*() value will be higher than if a restricted range is used, as discussed above. In order to compare the different *g*() values, we make an approximative correction by scaling the value with the fraction of the scale range observed. For example, the UPDRS data only covered 60% of the theoretical range and the MDS-UPDRS data covered 58%. On further inspection of the SD estimates after such adjustment, as seen in supplemental table 1, they range from 0.067 to 0.23. To take an example, the reason the MDS-UPDRS SD estimate is higher than the UPDRS estimate could be because the new questions added to the UPDRS questionnaire might have poorer separating properties than the established questions, especially for the de novo cohort that was being studied. The population studied in the UPDRS data was also further progressed and located in the middle of the scale, where the UPDRS scale was designed to be best at describing and separating patients.

### Pearson Residual: a Probability Weighted Residual

As seen in Fig. 2, the Pearson residuals seem to have a mean of 0 and a variance of approximately 1. The bias is low, and overall there are only small trends in the residuals, but especially the UPDRS model could be improved according to the results. However, it was not the purpose of this exercise to further develop the existing models but rather to make a fair comparison between model types. The model building could have resulted in a different final model if the BI approach had been used from the start. This is however a topic outside the scope of this work.

### General Discussion

In the present work, we have used cut-offs for the probit function driven by the standard normal distribution, and a normal distribution was also the choice for the mean-variance (*f*() − *g*()) function. One could imagine other ways of determining the cut-offs as well as choosing other distribution functions. For the former, it is possible to estimate the cut-off values at the expense of parsimony. This can also result in over-fitting with poor predictive performance. For the latter, other probability density functions than the standard normal could also be implemented, for example a *t*-distribution to allow for heavier tails. A Box-Cox transformed distribution might provide a better fit if the data distribution is skewed, as indicated by Hu *et al.* (9). In this paper, we focused on the normal distribution due to its simplicity and few assumptions regarding the data. This implementation showed an improvement in fit over OC and CV models in all investigated cases, save one, but this fact does not exclude that further refinements to the BI model implementation can be done.

For the one case where the BI model performed worse than the CV model, PANSS 1 data, further testing via residual modelling (not shown) indicated that the BI model could have been improved upon more than the corresponding CV model by adding a more flexible residual structure. Importance sampling with the expectation step only also gave a better fit than the CV model (not shown).

The main difference from the work by Ursino and Gasparini (8) and especially Hu *et al.* (9) is the implementation on several data sets, both rating and composite scale data, comparisons with more standard models, allowing random effects directly on the variability parameter and the implementation of Markov elements.

While rating scales with few categories often express the choice in words (no, mild, moderate, severe), a rating scale like the Likert already provide the integer numbers as guide for patients to guide their choice between no pain and worst possible pain. For this reason, it is likely that a scale like the Likert data is well described by the BI approach.

For a scale with a few numbers of scores, the gain of the BI approach is likely smaller. There is no general rule for when to switch to a CV model from an OC model, as demonstrated by the fact that the 11-category Likert data was modelled in both ways. Likewise, the gain of switching to a BI model cannot be stated by a definite rule. However, the number of scores (*n*) is one important aspect, where the advantages (parsimony, run times, parameter uncertainty) over OC models are expected to be larger as *n* increases. The advantage over CV models could depend on if there are many observations at the scale boundaries, but as exemplified with UPDRS and MDS-UPDRS, an advantage may be identified even when no data are close to scale limits.

In many cases, there are competing scales for assessing the same disease, e.g. Parkinson’s disease. The latent variable could serve as a link between such scales so that translation between scales is made possible. This could be helpful when pooling data for combined analysis. One example of such a translation is a recent model for nicotine craving data where both a 4-category scale and a visual analogue scale with 101 categories were measured (31).

## CONCLUSIONS

The bounded integer model provides a good description of rating and composite scale data, both in terms of fit and performance in external validation. It has shown better fit and performance in external validation to multiple data sets than models treating the same data as either ordered categorical or a continuous variable. Simulations from the model will provide real-life-like data that does not need rounding/truncation and/or transformation. Also, Markov elements can easily be added to the model.

## Electronic Supplementary Material

ESM 1 (DOCX 24.1 kb)

ESM 2 (MOD 3 kb)

ESM 3 (MOD 18 kb)

ESM 4 (DOCX 15.3 kb)
