# Supplementary material for: A Bounded Integer Model for Rating and Composite Scale Data
Source: AAPS J. 2019 Jun 6;21(4):74. doi: 10.1208/s12248-019-0343-9 (PMC6554249; doi:10.1208/s12248-019-0343-9)
Supplement: Supplementary file 1 — (DOCX 24.1 kb) [file 12248_2019_343_MOESM1_ESM.docx]

Supplemental table 1. Parameter estimates for bounded integer models

| BI Model | Parameter | Typical value (SE) | IIV variance (SE) | IOV variance (SE) |
| --- | --- | --- | --- | --- |
| MDS-UPDRS motor | Base [-] | -0.988 (0.016) | 0.10 (0.0061) |  |
|  | SD [-] | 0.148 (0.0028) | 0.052 (0.015) |  |
|  | Disease progression [month^-1^] | 0.0101 (0.00070) | 5.3E-5 (1.5E-5) |  |
|  | Drug effect [-] | 0.125 (0.016) | 0.021 (0.0068) |  |
|  |  |  |  |  |
| PANSS 1 | Baseline PANSS score in Phase II [-] | -0.382 (0.048) | 0.039 (0.0040) |  |
|  | Maximum placebo effect (P_max_) [-] | 0.161 (0.015) | 0.080 (0.010) |  |
|  | Time course of placebo effect [days] | 17.7 (1.0) | - |  |
|  | SD [-] | 0.0673 (0.0041) | 0.26 (0.029) |  |
|  | Asenapine AUC_50_ [μg*h/L] | 448 (81) | 17 (14) |  |
|  | Maximum asenapine effect (E_max_) [-] | 0.0513 (0.030) | - |  |
|  | Power parameter in Weibull function [-] | 1.21 (0.034) | - |  |
|  | Proportional difference in SD for patients studied in the United States [-] | 0.512 (0.085) | - |  |
|  | Proportional difference in baseline PANSS score for chronic patients [-] | -0.0477 (0.033) | - |  |
|  | Proportional difference in P_max_ for phase III patients [-] | 0.745 (0.14) | - |  |
|  | Baseline PANSS score in Phase III [-] | -0.434 (0.040) | - |  |
|  | Proportional difference in SD for patients not hospitalized [-] | -0.00212 (0.052) | - |  |
|  |  |  |  |  |
| ADAS-Cog | Rate of change of ADAS-Cog due to disease progression [year^-1^] | 0.176 (0.017) | - |  |
|  | Intercept of linear model of bADAS-bMMSE [] | 1.58 (0.070) | 0.053 (0.0037) |  |
|  | Slope of linear model of bADAS-bMMSE [] | -0.0980 (0.0030) | 7.1E-5 (1.1E-5) |  |
|  | SD [-] | 0.172 (0.0025) | - |  |
|  | Baseline ADAS-Cog [-] | 3.05 (0.26) | - |  |
|  | Age effect [-] | -2.01 (0.62) | - |  |
|  | *APOE* ε4 effect [-] | 1.36 (0.12) | - |  |
|  | Sex effect (male) [-] | 0.889 (0.083) | - |  |
|  |  |  |  |  |
| PANSS 2 | Baseline PANSS score [-] | -0.435 (0.093) | 0.026 (0.0061) |  |
|  | SD [-] | 0.0760 (0.0026) | 0.25 (0.028) |  |
|  | Maximum placebo effect (P_max_) [-] | 0.167 (0.011) | 0.15 (0.019) |  |
|  | Proportional difference in baseline PANSS score for chronic patients [-] | -0.0586 (0.11) | - |  |
|  | Time course of placebo effect [days] | 24.8 (2.5) | - |  |
|  | Paliperidone AUC_50_ [μg*h/L] | 5530 (600) | 1.7 (0.36) |  |
|  | Maximum paliperidone effect (E_max_) [-] | 9.47 (1.3) | - |  |
|  | Power parameter in Weibull function [-] | 1.05 (0.0043) | - |  |
|  | Proportional difference in SD for patients not hospitalized [-] | 0.0807 (0.039) | - |  |
|  | Proportional difference in SD for patients studied in the United States [-] | 0.547 (0.051) | - |  |
|  | Proportional difference in P_max_ for phase III patients [-] | 0.68 (Fixed) | - |  |
|  |  |  |  |  |
| UPDRS motor | K_eo_ [h^-1^] | 0.449 (0.060) | 0.71 (0.18) | 0.20 (0.019) |
|  | E_0_ [-] | 0.0546 (0.034) | 0.050 (0.0086) | - |
|  | C_50_ carbidopa [ng/mL] | 1060 (47) | 0.080 (0.013) | 0.021 (0.0026) |
|  | γ [-] | 0.100 (3.0E-5) | 0.95 (0.29) | - |
|  | E_max_ [-] | -0.611 (0.022) | 0.031 (0.0067) | - |
|  | C_50_ benserazide [ng/mL] | 1130 (76) | 0.080 (0.013) | 0.021 (0.0026) |
|  | SD [-] | 0.0708 (0.0019) | 0.086 (0.025) | - |
|  | Correlation [η_keo,_ η_E0_] | - | 0.146 (0.038) | - |
|  |  |  |  |  |
| Likert (9 parameters) | Base [-] | 0.261 (0.048) | 0.22 (0.040) |  |
|  | Maximum placebo effect (P_max_) [-] | -0.548 (0.15) | 1.0 (0.22) |  |
|  | Half-life [days] | 72.1 (24) | 1.3 (0.42) |  |
|  | SD [-] | 0.229 (0.0090) | - |  |
|  | Effect of paracetamol [-] | 0.198 (0.031) | - |  |
|  | PM [-] | 0.531 (0.035) | - |  |
|  |  |  |  |  |
| Likert (14 parameters) | Base [-] | 0.288 (0.025) | 0.18 (0.036) |  |
|  | Maximum placebo effect (P_max_) [-] | -0.562 (0.089) | 0.81 (0.20) |  |
|  | Half-life [days] | 38.3 (3.1) | 1.1 (0.11) |  |
|  | SD start [-] | 0.0437 (0.0024) | 0.98 (0.091) |  |
|  | Effect of paracetamol [-] | 0.127 (0.025) | - |  |
|  | SD end [-] | 0.0222 (0.0012) | 0.98 (0.091) |  |
|  | PM end [-] | 0.432 (0.033) | - |  |
|  | PM start [-] | 0.266 (0.023) | 1.9 (0.16) |  |
|  | Markov half-life [days] | 0.490 (Fixed) | 1.9 (0.16) |  |

UPDRS, Unified Parkinson’s Disease Rating Scale; MDS-UPDRS, Movement Disorder Society - Unified Parkinson’s Disease Rating Scale; ADAS-Cog, Alzheimer’s Disease Assessment Scale-Cognitive; PANSS, Positive and Negative Syndrome Scale; IIV, inter-individual variability; IOV, inter-occasion variability; SE, standard error; SD, standard deviation; AUC, area under plasma concentration-time profile; K_eo_, rate constant; E_0_, effect at baseline; C_50_, concentration required to reach 50% of maximal effect; E_max_, maximal effect; bADAS, baseline ADAS-Cog status; bMMSE, baseline Mini-Mental State Examination; *APOE* ε4, apolipoprotein ε4; γ, slope.
