# Supplementary material for: A Bounded Integer Model for Rating and Composite Scale Data
Source: AAPS J. 2019 Jun 6;21(4):74. doi: 10.1208/s12248-019-0343-9 (PMC6554249; doi:10.1208/s12248-019-0343-9)
Supplement: Supplementary file 4 — (DOCX 15.3 kb) [file 12248_2019_343_MOESM4_ESM.docx]

For a given latent variable U, we define a Standardized Transformed Score (STS) on [0,1]:

$$STS=\frac{k}{n}, k=0,\ldots,n$$

if and only if:

$$a_{k}\leq U\leq a_{k+1}$$

where:

$$a_{k}= \left\{ \begin{aligned} 0 if k=0 \\ 1 if k=1 \\ \frac{k-0.5}{2} otherwise \end{aligned} \right.$$

Define p, the individual prediction:

$$probit\left( U \right)=p$$

And assume a standard normal distribution:

$$p\sim N(f(,{}_{i,f},t,X_{i,f}),g(,{}_{i,g},t,X_{i,g}))$$

Where represents fixed effects parameter vector, ${}_{i}$ represents random effects variable vector for individual *i*, t represents time, represents variance of latent variable and X_i_ represents covariate vector of individual *i*.

The probability of observing a category STS = k/n is defined through:

$$P_{i,j}\left( k \right)=\phi\left( \frac{Z_{\frac{k}{n}}-f(,{}_{i,f},t,X_{i,f})}{g(,{}_{i,g},t,X_{i,g})} \right)-\phi\left( \frac{Z_{\frac{k-1}{n}}-f(,{}_{i,f},t,X_{i,f})}{g(,{}_{i,g},t,X_{i,g})} \right)$$

With special cases for *k=1* and *k=n:*

$$P_{i,j}\left( 1 \right)=\phi\left( \frac{Z_{\frac{1}{n}}-f(,{}_{i,f},t,X_{i,f})}{g(,{}_{i,g},t,X_{i,g})} \right)$$

$$P_{i,j}\left( n \right)=1-\phi\left( \frac{Z_{\frac{n-1}{n}}-f(,{}_{i,f},t,X_{i,f})}{g(,{}_{i,g},t,X_{i,g})} \right)$$

where φ is the cumulative distribution function of the normal distribution and Z_k/n_ and Z_(k-1)/n_ are the Z-scores defined by the probit function.

To define the likelihood function, we specify the inter-individual variability through the variance-covariance matrix $\Omega$:

$$\eta_{i}\sim N(0,\Omega)$$

And the observation vector of individual *i*:

$$Y_{i}=\left\{ Y_{i,1},\ldots,Y_{i,j} \right\}$$

Then we arrive at the marginal likelihood for individual *i*:

$$L(\theta,\Omega,\sigma|Y_{i})=\int\prod_{j}^{n_{i}} l\left( Y_{i,j} | \eta_{i} \right)f\left( \eta_{i} \right)d\eta$$

Where $l\left( Y_{i,j}=k | \eta_{i} \right)$ is given by the probability P_i,j_(k) described above.
